# Gradient-induced voltages on 12-lead ECGs during high-duty-cycle MRI sequences and a theoretically-based method to remove them

**DOI:** 10.1186/1532-429X-17-S1-P243

**Published:** 2015-02-03

**Authors:** Shelley H Zhang, Zion T Tse, Charles L Dumoulin, Ronald D Watkins, Wei Wang, Jay Ward, Raymond Y Kwong, William G Stevenson, Ehud J Schmidt

**Affiliations:** Engineering, The University of Georgia, Athens, GA USA; Radiology, Cincinnati Children’s Hospital Medical Center, Cincinnati, OH USA; Radiology, Stanford University, Stanford, CA USA; E-Trolz Inc, Andover, MA USA; Cardiology, Brigham and Women’s Hospital, Boston, MA USA; Radiology, Brigham and Women’s Hospital, Boston, MA USA

## Background

An MRI-compatible 12-lead ECG platform, equipped with MRI-gradient induced-voltage removal hardware and magneto-hydrodynamic voltage removal software [1], was previously applied to physiological monitoring and synchronization of cardiac imaging of patients inside MRI. This approach had limited success for high-duty-cycle [(total Gradient-ramp-time per R-R)/(R-R time) >20%] MRI sequences, such as Steady State Free Precession (SSFP), Short-TR Gradient Echo (GRE), and Short-TR Fast Spin Echo. The study objective is to measure and develop a method to remove gradient-induced voltages on 12-lead ECGs during high-duty-cycle MRI sequences.

## Methods

**Figure 1:**
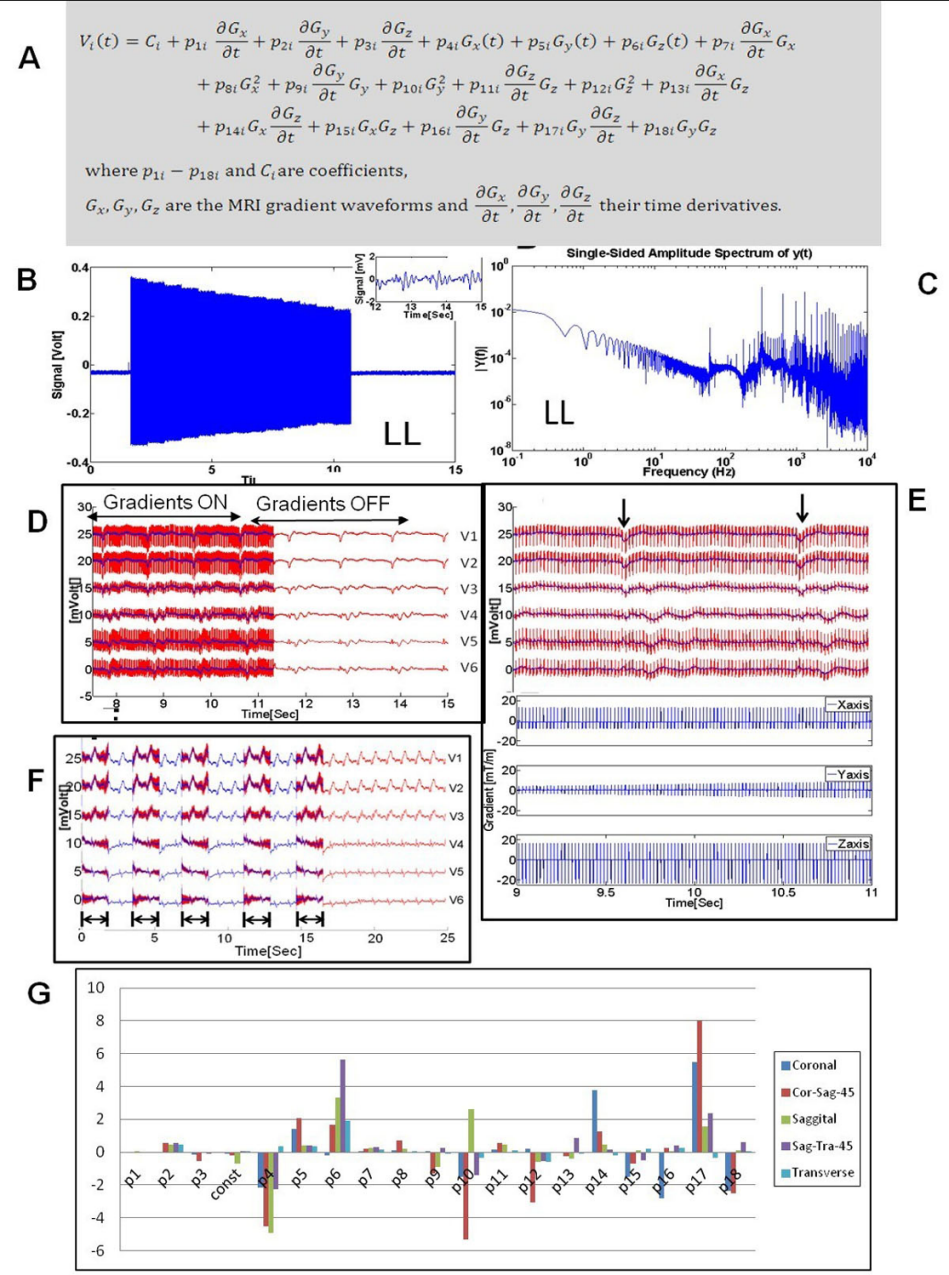
(A) Theoretically derived 19-parameter equation for the gradient-induced voltage at each ECG Electrode (i). (B) Measured induced voltage at the Left Leg (LL) during and SSFP sequence (TR/TE=3.09/1.32ms). Magnitude is 0.7 Volts. Inset shows real ECG when MRI was not pulsing. (C) Frequency spectrum of induced-voltage on LL during SSFP sequence. Spectral peaks are at 60Hz and a harmonics of (1/TR) frequency ~320 Hz. (D) ECG traces V1-V6 recorded during a training GRE sequence (TR/TE=20/1.97ms), which consisted of intermittent imaging (Gradient ON) and non-imaging (Gradient OFF) segments. The non-imaging segment was then subtracted from the traces acquired during imaging ("corrupted ECGs", red lines), and the resulting traces fitted to the theoretical equation, in order to compute its coefficients P_11- P18i_ and C_i._ The calculated induced-voltages were then removed from the corrupted ECGs, resulting in restored ECGs (blue line) that were very similar to the true ECG. (E) Zoomed view of imaging-segment in (D), showing corrupted (red) and restored (blue lines) traces. Bottom Section showing gradient waveforms along x, y, and z directions recorded during this time. Black arrows highlight QRS complexes, which are easily observed in the restored traces. (F) during a full-resolution multi-slice SSFP sequences, the recorded ECG traces (red line) from 5 parallel slices were cleaned (blue line) in real-time using the equation, using coefficients calculated from a prior single-slice accelerated (training) sequence. (G) The 19-fit parameters for the V6 electrode for the same SSFP sequence acquired along multiple directions in a single volunteer; Coronal, 45 degrees oblique between Coronal and Sagittal, Sagittal, 45 degrees oblique between Sagittal and Transverse, Transverse.

A modification of the hardware developed in [1] enabled measuring the gradient-induced voltage over a 24 kHz frequency-range and +/-10V, together with the x, y, and z gradient waveforms. ECGs were measured in 9 volunteers at 3T (Siemens Skyra). A theoretical equation for the gradient-induced voltages on each ECG electrode (V_i_, where i=1,2,3..9 was derived, based on Maxwell's equations [2] and concomitant fields [3] [Fig. 1A]. It includes 1st and 2nd order gradient waveform terms and estimates induced voltages even on ECG electrodes positioned farthest from magnet iso-center, such as the limb leads.

The 19 equation coefficients were obtained during 6-8 second training sequences, consisting of highly accelerated (3-4sec, GRAPPA=6-8) versions of each sequence, followed by non-imaging segments. The non-imaging segments were used to obtain the shape of the true ECG traces over the entire R-R cycle ("template"). This template was subtracted from ECG traces acquired during imaging, resulting in the net gradient-induced voltages, which was then fit to the equation, providing the 19 coefficients for each electrode.

Multi-slice imaging was then performed, with real-time subtraction of the gradient-induced voltages from each acquired ECG trace, utilizing the computed V_i_.

## Results

Measured limb-lead ECG voltages during SSFP imaging, with the heart at iso-center (Fig. 1B), were 0.7-1.0 Volt PTP, with frequency components up to 20 KHz (Fig. 1C). Applying the equation for gradient-induced voltage removal during GRE (Fig. 1D-E), and multi-slice SSFP imaging (Fig. 1F), resulted in a normalized-difference between the recovered ECG trace and the true ECG of <20%. Equation coefficients varied by subject, sequence, sequence parameters, and slice orientation (Fig. 1G).

## Conclusions

An equation was derived for the strong gradient-induced voltages observed in 12-lead ECGs during high-duty-cycle MRI sequences. A rapid training sequence permitted computing equation-coefficients, followed by real-time gradient-induced voltage removal during imaging.

## Funding

NIH U41-RR019703, R03-EB013873-01A1, AHA 10SDG261039.
